# Supplementary material for: Diffusion MRI head motion correction methods are highly accurate but impacted by denoising and sampling scheme
Source: Hum Brain Mapp. 2024 Jan 30;45(2):e26570. doi: 10.1002/hbm.26570 (PMC10826632; doi:10.1002/hbm.26570)
Supplement: Supplementary file 1 — TABLE S1. Statistical comparisons of denoising, scheme type, number of directions, denoising method, and motion prevalence on RMSE of estimated motion. TABLE S2. Statistical comparison of Eddy and SHORELine in shelled schemes. The outcome measure was the difference in error (RMSE) between Eddy and SHORELine (Eddy–SHORELine); the model included main effects of denoising, motion prevalence in the input data, and shell scheme; interactions of these main effects were modeled as well. TABLE S3. Statistical comparisons of image smoothness between Eddy and SHORELine. The outcome measure was the difference in image smoothness (FWHM) between Eddy and SHORELine (Eddy–SHORELine); the model included main effects of denoising, motion prevalence in the input data, and sampling scheme. TABLE S4. Statistical comparisons of image quality between Eddy and SHORELine. The outcome measure was the difference in image quality (NDC) between Eddy and SHORELine (Eddy–SHORELine); the model included main effects of denoising, motion prevalence in the input data, and shell scheme. FIGURE S1. Empirical motion parameters in large‐scale datasets. QSIPrep outputs from the ABCD data processing and data from the public release of Healthy Brain Network (HBN; Richie‐Halford et al., 2022) study were used to obtain automated quality control (QC) metrics for ABCD and HBN datasets, respectively. The distribution of maximum translation (in mm) and rotation (in degrees) values are depicted for both datasets. The empirical motion parameters range widely, such that maximum translation = [0.22 9.09] and maximum rotation = [0.10 6.60] for ABCD (top row), and maximum translation = [0.09 20.78] and maximum rotation = [0.01 10.61] for HBN (bottom row). [file HBM-45-e26570-s001.docx]

**Diffusion MRI Head Motion Correction Methods are Highly Accurate but Impacted by Denoising and Sampling Scheme**

Matthew Cieslak^1,2*^, Philip A. Cook^3,4^, Golia Shafiei^1,2^, Tinashe M. Tapera^1,2^, Hamsanandini Radhakrishnan^1,2^, Mark Elliott^3^, David R. Roalf^2^, Desmond J. Oathes^2^, Dani S. Bassett,^2,4,5,6,7,8^ M. Dylan Tisdall^2^, Ariel Rokem^9^, Scott T. Grafton^10^,

& Theodore D. Satterthwaite^1,2,11,*^

^1^ Lifespan Informatics and Neuroimaging Center, University of Pennsylvania Perelman School of Medicine

Departments of Psychiatry,^2^ Radiology,^3^ Neurology,^4^ Bioengineering,^5^ Physics & Astronomy,^6^ Electrical and Systems Engineering,^7^ University of Pennsylvania Perelman School of Medicine

^8^Sante Fe Institute

^9^ Department of Psychology and the eScience Institute, University of Washington

^10^ Department of Psychological and Brain Sciences, University of California Santa Barbara

^11^ Penn-CHOP Lifespan Brain Institute

*Please direct correspondence to:

Matt Cieslak: Matthew.Cieslak@pennmedicine.upenn.edu

Theodore D. Satterthwaite: sattertt@pennmedicine.upenn.edu

Richards Medical Labs, 5th Floor, Pod A

3700 Hamilton Walk

Philadelphia, PA 19104

**SUPPLEMENTARY MATERIAL**

**Supplementary Table 1.** Statistical comparisons of denoising, scheme type, number of directions, denoising method, and motion prevalence on RMSE of estimated motion.


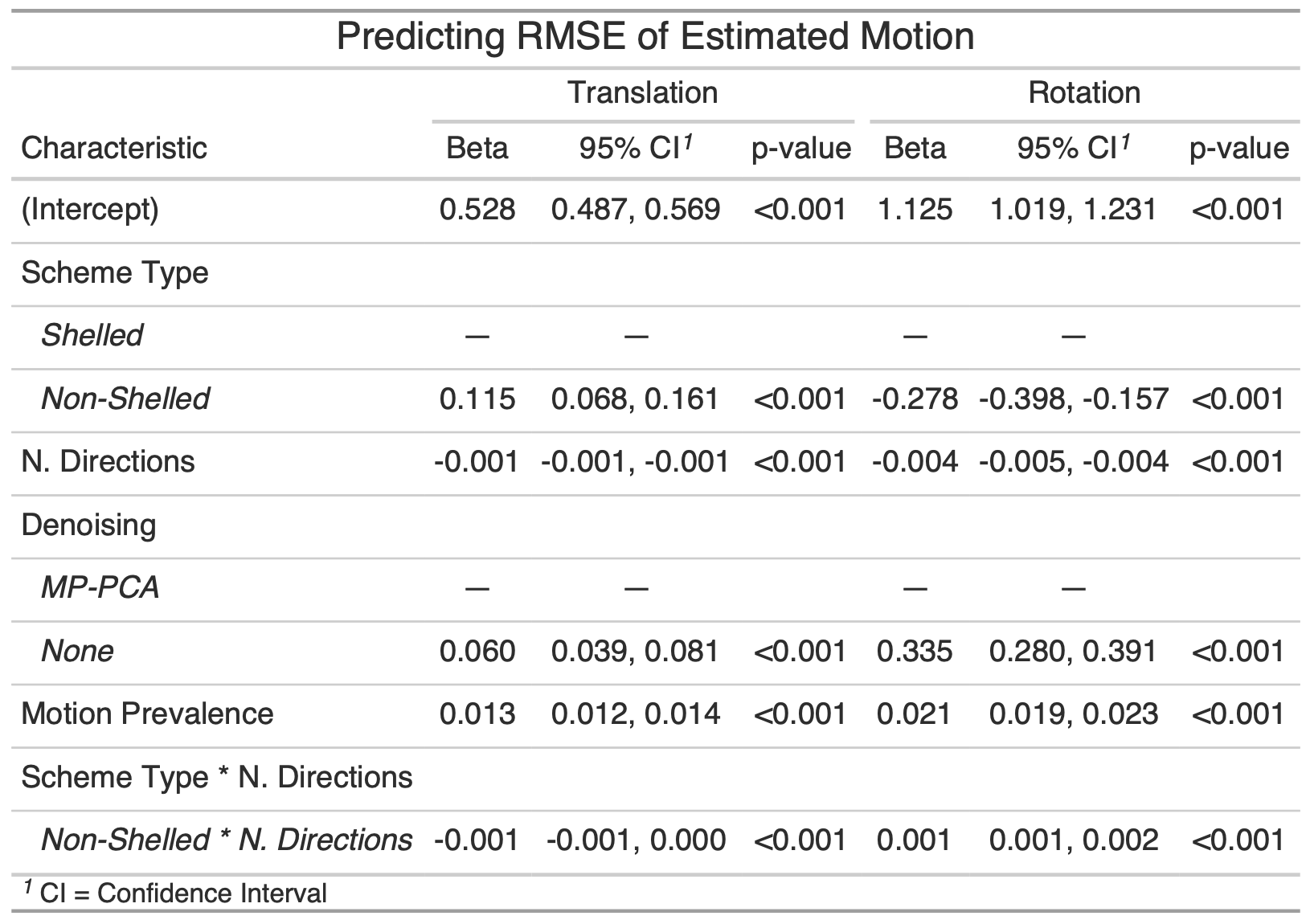


**Supplementary Table 2.** Statistical comparison of Eddy and Shoreline in shelled schemes. The outcome measure was the difference in error (RMSE) between Eddy and Shoreline (Eddy-Shoreline); the model included main effects of denoising, motion prevalence in the input data, and shell scheme; interactions of these main effects were modeled as well.

**
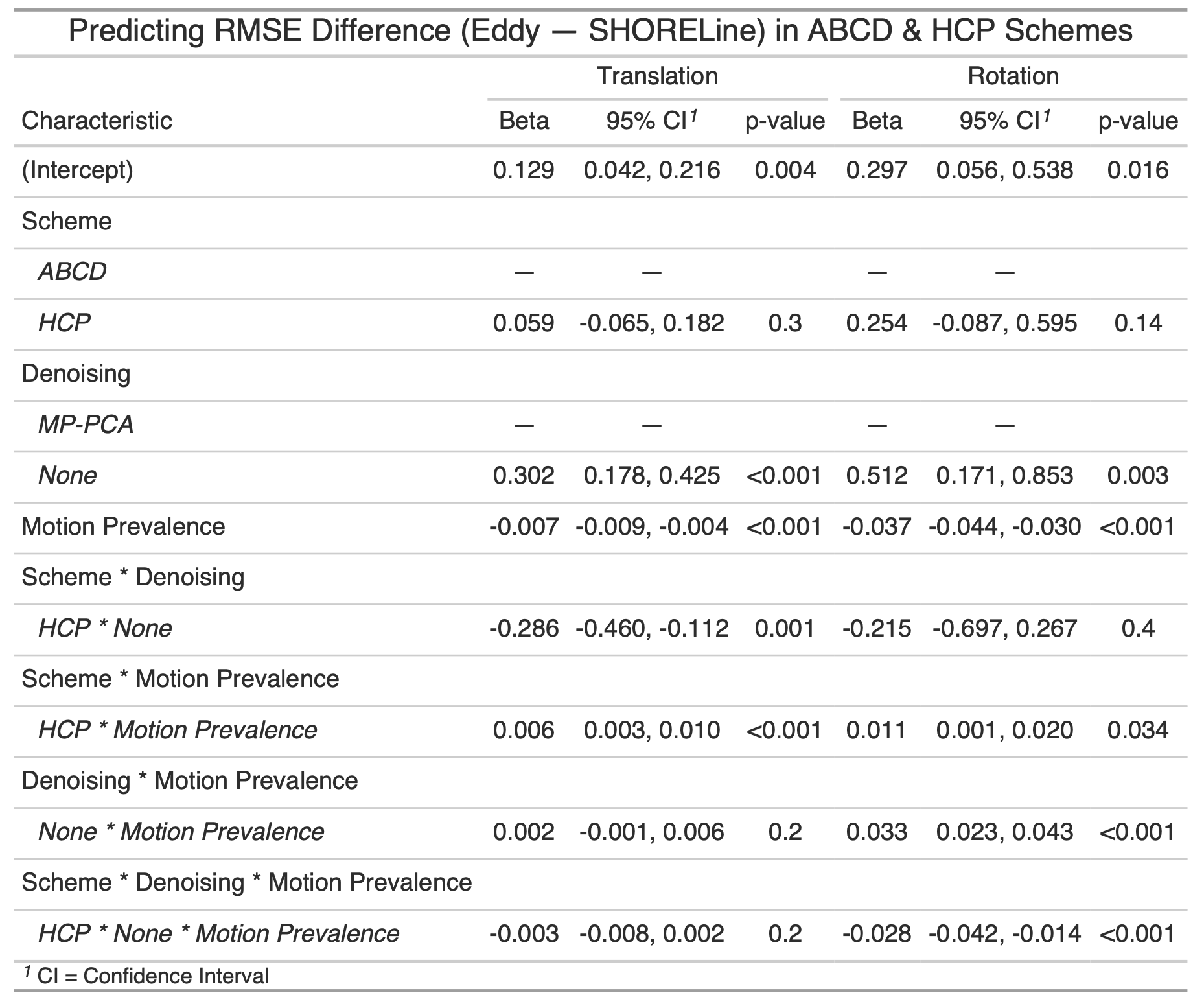
**

**Supplementary Table 3**. Statistical comparisons of image smoothness between Eddy and Shoreline. The outcome measure was the difference in image smoothness (FWHM) between Eddy and Shoreline (Eddy-Shoreline); the model included main effects of denoising, motion prevalence in the input data, and sampling scheme.


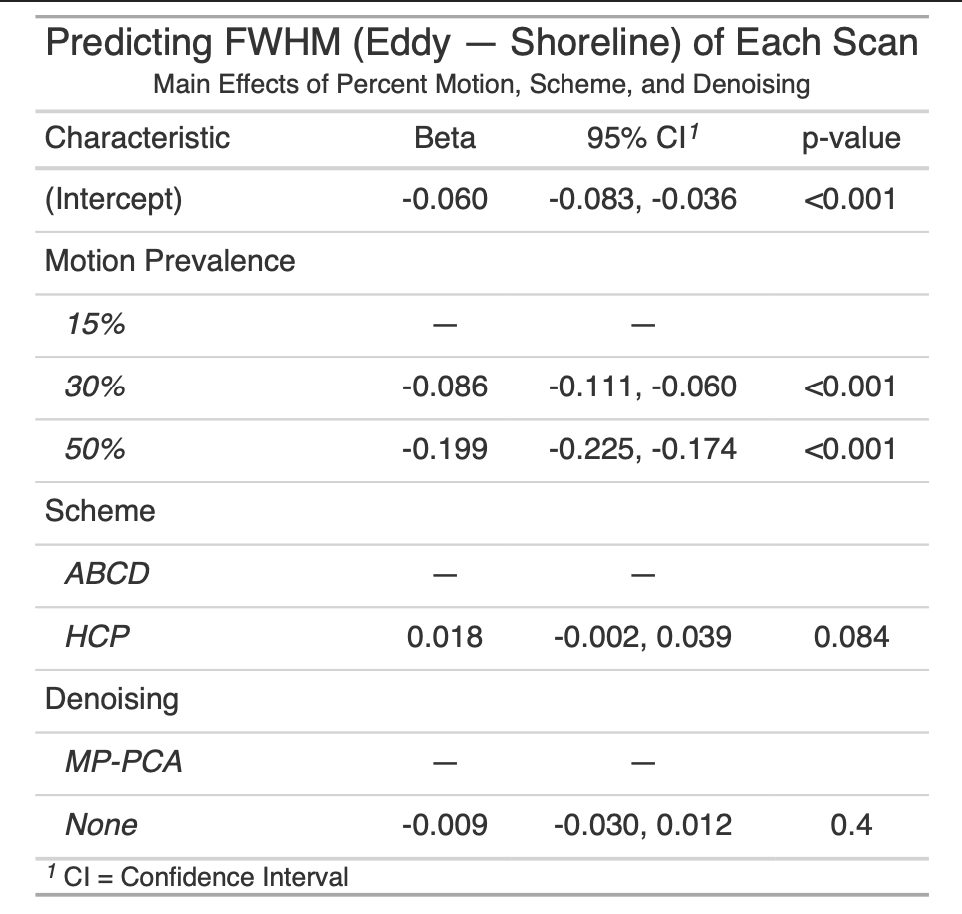


**Supplementary Table 4.**  Statistical comparisons of image quality between Eddy and Shoreline. The outcome measure was the difference in image quality (NDC) between Eddy and SHORELine (Eddy-Shoreline); the model included main effects of denoising, motion prevalence in the input data, and shell scheme.


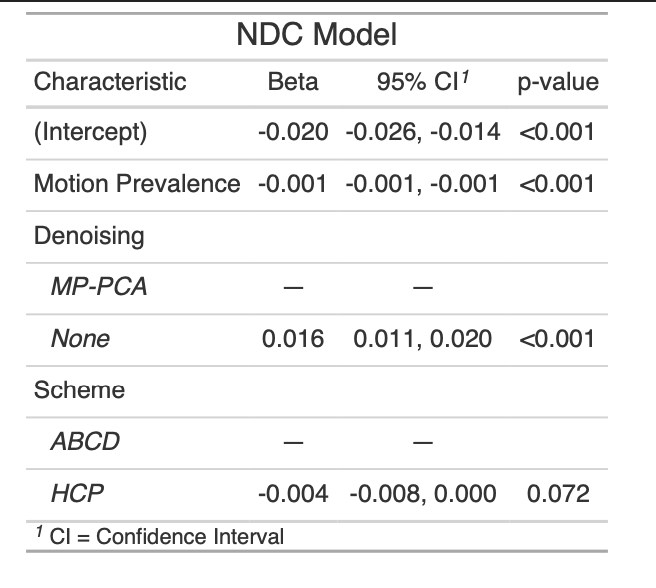


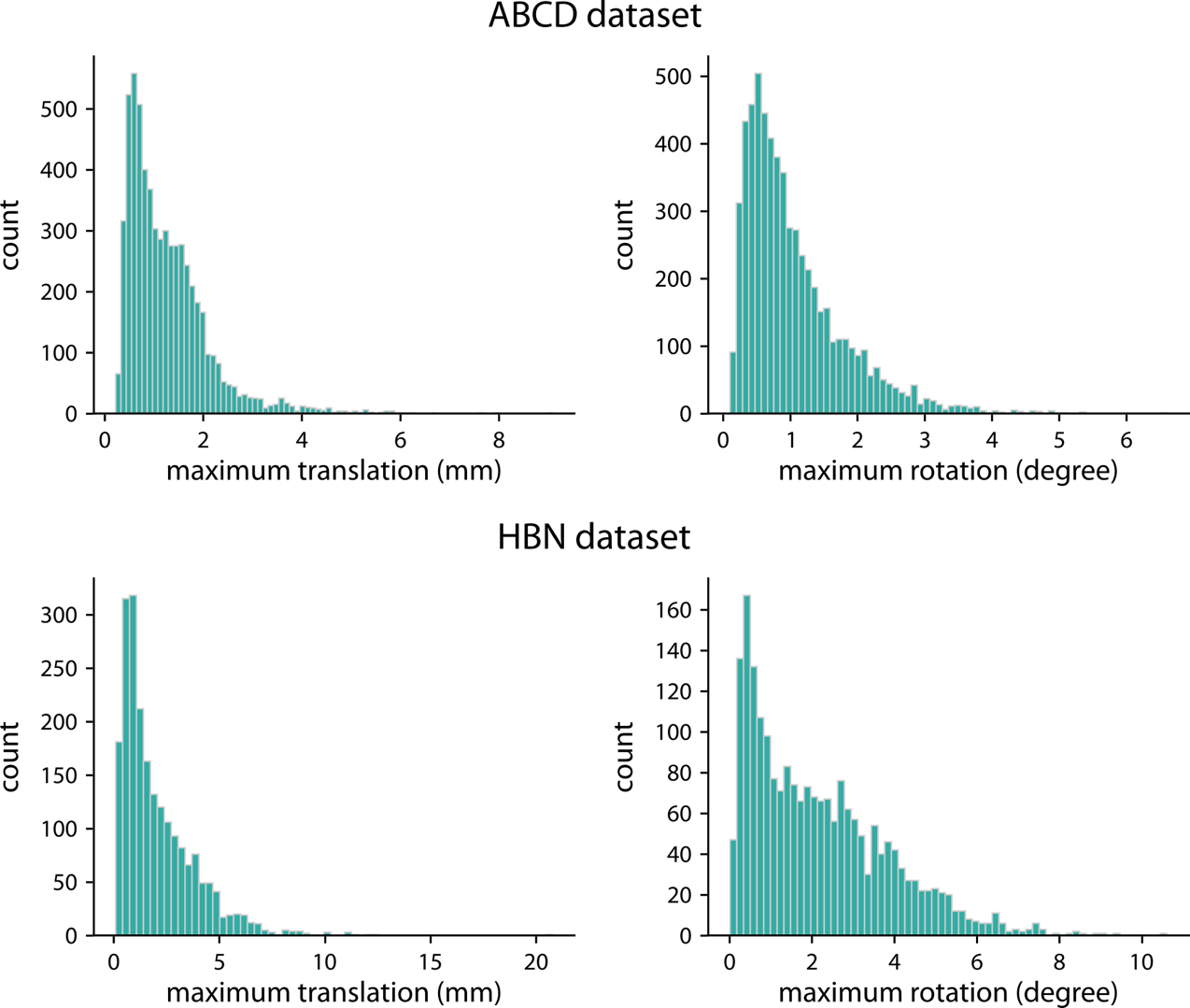


**Figure S1. Empirical motion parameters in large-scale datasets.** QSIPrep outputs from the ABCD data processing and data from the public release of Healthy Brain Network (HBN; Richie-Halford et al., 2022) study were used to obtain automated quality control (QC) metrics for ABCD and HBN datasets, respectively. The distribution of maximum translation (in millimeters; *mm*) and rotation (in degrees) values are depicted for both datasets. The empirical motion parameters range widely, such that maximum translation = [0.22 9.09] and maximum rotation = [0.10 6.60] for ABCD (top row), and maximum translation = [0.09 20.78] and maximum rotation = [0.01 10.61] for HBN (bottom row).
